# Preventing cell death with a ‘check valve' in mitochondrial complex I?

**DOI:** 10.1038/cddis.2016.71

**Published:** 2016-03-31

**Authors:** E Fontaine, D Detaille, G Vial

**Affiliations:** 1 University Grenoble Alpes, LBFA, Grenoble F-38000, France; 2 INSERM, U1055, Grenoble F-38000, France; 3 INSERM U1045, LIRYC (L'Institut de Rythmologie et Modélisation Cardiaque), Pessac F-33600, France; 4 INSERM U1060, Laboratoire CarMeN, Université Lyon 1, INRA 1235, INSA de Lyon, Facultés de Médecine Charles Mérieux Lyon-Sud et Rockfeller, Lyon F-69003, France

As the discovery that some mitochondrial proteins located between the inner and outer mitochondrial membranes promoted cell death once released into the cytosol, mitochondria have been acknowledged as key organelles in programmed cell death.^1^ One of the mechanisms triggering the release of mitochondrial pro-apoptotic proteins is the opening of an inner membrane channel named the permeability transition pore (PTP).^2^ Among the many parameters inducing PTP opening, oxidative stress is known to promote PTP opening in intact cells.^3^ The PTP is a multi-protein complex composed of a core (the channel itself) regulated by other proteins, including cyclophilin D that makes PTP opening easier by binding with the rest of the pore.^4^ The amount of cyclophilin D varies depending on tissues, which explains why drugs detaching cyclophilin D from the pore (e.g., Cyclosporine A) are less effective at PTP inhibition in tissues with low amounts of cyclophilin D.^5^ In all the tissues tested so far, the inhibition of respiratory chain complex I has been shown to inhibit PTP opening, either spontaneously (in tissues with low amount of cyclophilin D) or once cyclophilin D had been detached from the pore.^5^ Because both complex I inhibition and cyclophilin D detachment require phosphate to inhibit PTP opening, a model has been proposed in which the number of binding sites for phosphate depends on complex I activity, while the binding of phosphate is prevented by cyclophilin D.^5^

Initially, the regulatory effect of complex I activity on PTP opening was revealed by using mitochondrial poisons known to inhibit complex I such as rotenone or piericidine.^6^ However interesting it was from a molecular point of view, the use of such poisons for PTP regulation was obviously inconceivable *in vivo*. Yet, the recognition that the widely prescribed anti-diabetic drug metformin that partly inhibits complex I^7^ also inhibited PTP opening^8^ made it possible to consider complex I as a realistic target for PTP regulation *in vivo*.

Complex I is the first of the three proton pumps that builds up the protonmotive force by coupling redox reactions to a vectorial transfer of protons. Normally, complex I catalyzes the transfer of electrons from NADH+H^+^ to the ubiquinone pool. However, complex I is a reversible enzyme that can consume the protonmotive force to transfer electrons from the ubiquinol pool to NAD^+^. Both during the forward and the reverse electron transfer, some of them can escape the normal pathway to reduce oxygen in superoxide.^9^ By affecting the electron flow in complex I, complex I inhibitors such as rotenone and metformin increase and decrease the electron leak (i.e., superoxide production) driven by the forward and reverse electron transfers, respectively.^9^

In our recently published work in Cell Death Discovery,^10^ we have reported a hitherto unrecognized situation in which superoxide production driven by the reverse electron transfer is dramatically reduced, without any effect on oxygen consumption of intact cells, on cell energy status and on isolated complex I activity. This unexpected behavior was observed after the incubation of human endothelial cells in the presence of Imeglimin, a new oral glucose-lowering agent.^11^ By inhibiting superoxide production driven by the reverse electron transfer (presumably by inhibiting the reverse electron transfer) with no inhibition of the forward electron transfer, Imeglimin acted as a check valve on complex I. As for now, the mechanism through which Imeglimin inhibits superoxide production driven by the reverse electron transfer remains unknown, but it is probably unconventional as all the other drugs known to do this also inhibit the forward electron transfer. Most importantly, not only does Imeglimin inhibit superoxide production driven by the reverse electron transfer^10^ but it also prevents PTP opening and subsequent cell death induced by exposure to high glucose or oxidizing agent *tert*-Butyl hydroperoxide.^10^

Using another cell line and another model to induce PTP opening-induced cell death, we recently observed that experimental conditions preventing oxidative stress (incubation in the absence of oxygen or incubation in the presence of antioxidant *N*-acetyl-cysteine) prevented PTP opening and subsequent cell death induced by the removal of energy substrates.^12^ Interestingly, metformin—which is not an antioxidant but prevents superoxide production driven by the reverse electron transfer^13^—also prevented PTP opening and subsequent cell death.^12^ This strongly suggests that such a particular superoxide production is mandatory for permanent PTP opening and thus for this type of cell death. We therefore propose a hypothetical model (Figure 1) in which the superoxide production driven by the reverse electron transfer specifically promotes PTP opening. This could be due to a conformational change in complex I that in turn may make the PTP more sensitive to superoxide.

Because up to now, all the compounds able to prevent superoxide production driven by the reverse electron transfer have been shown to inhibit PTP opening,^6, 8, 12^ we suggest that preventing the reverse electron transfer would be sufficient to inhibit PTP opening. The toxicity of rotenone and piericidin precludes their clinical use. In contrast, metformin is widely prescribed and could be used to prevent PTP opening. However, rare cases of metformin poisoning (leading to lactic acidosis) have been reported. Our results suggest that this risk should disappear if using drugs that only inhibit the reverse electron transfer through complex I.

## Figures and Tables

**Figure 1:**
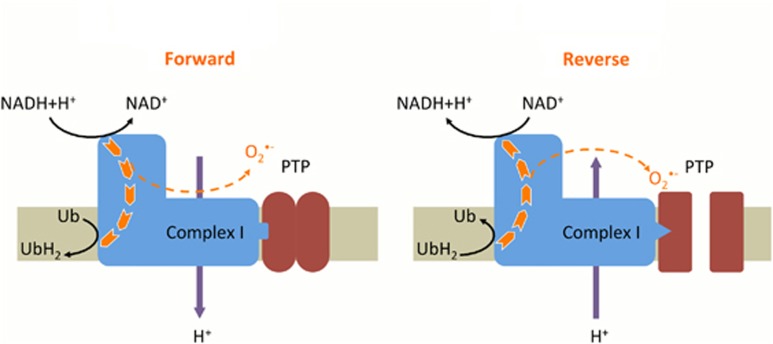
Hypothetical model in which reverse electrons flow through complex I promotes PTP opening. Superoxide is generated during electron transfer through complex I, both during the forward and the reverse electron transfer. We hypothesized that reverse electron transfer requires or induces conformational changes in complex I, which in turn makes the PTP more sensitive to oxidative stress, thereby promoting PTP opening
